# Biomolecule and Bioentity Interaction Databases in Systems Biology: A Comprehensive Review

**DOI:** 10.3390/biom11081245

**Published:** 2021-08-20

**Authors:** Fotis A. Baltoumas, Sofia Zafeiropoulou, Evangelos Karatzas, Mikaela Koutrouli, Foteini Thanati, Kleanthi Voutsadaki, Maria Gkonta, Joana Hotova, Ioannis Kasionis, Pantelis Hatzis, Georgios A. Pavlopoulos

**Affiliations:** 1 Institute for Fundamental Biomedical Research, Biomedical Sciences Research Center “Alexander Fleming”, 16672 Vari, Greece; zafeiropoulou@fleming.gr (S.Z.); karatzas@fleming.gr (E.K.); mikaela.koutrouli@cpr.ku.dk (M.K.); fotinithanati@gmail.com (F.T.); voutsadaki@fleming.gr (K.V.); mgkonta97@gmail.com (M.G.); hotova@fleming.gr (J.H.); gkasionis2@gmail.com (I.K.); hatzis@fleming.gr (P.H.); 2 Novo Nordisk Foundation Center for Protein Research, University of Copenhagen, 2200 Copenhagen, Denmark; 3 Center for New Biotechnologies and Precision Medicine, School of Medicine, National and Kapodistrian University of Athens, 11527 Athens, Greece

**Keywords:** biomedical networks, associations, biological interactions, network biology, databases, data integration

## Abstract

Technological advances in high-throughput techniques have resulted in tremendous growth of complex biological datasets providing evidence regarding various biomolecular interactions. To cope with this data flood, computational approaches, web services, and databases have been implemented to deal with issues such as data integration, visualization, exploration, organization, scalability, and complexity. Nevertheless, as the number of such sets increases, it is becoming more and more difficult for an end user to know what the scope and focus of each repository is and how redundant the information between them is. Several repositories have a more general scope, while others focus on specialized aspects, such as specific organisms or biological systems. Unfortunately, many of these databases are self-contained or poorly documented and maintained. For a clearer view, in this article we provide a comprehensive categorization, comparison and evaluation of such repositories for different bioentity interaction types. We discuss most of the publicly available services based on their content, sources of information, data representation methods, user-friendliness, scope and interconnectivity, and we comment on their strengths and weaknesses. We aim for this review to reach a broad readership varying from biomedical beginners to experts and serve as a reference article in the field of Network Biology.

## 1. Introduction

Technological advances in various high-throughput techniques in the last decade have led to an explosion of information about how biomolecules operate and function in living systems. Microarray and RNAseq technologies, for example, provide insights into gene expression levels and changes. scRNAseq technology organizes cells into groups based on their gene expression profiles and mass spectrometry is used for the identification of proteins based on their molecular weights and mass-to-charge ratio. Nuclear magnetic resonance (NMR) and X-ray crystallography are used for the determination of 3D protein structures while genome sequencing can provide insights about genetic variations, polymorphisms, chromatin structure and state, and species identification. Finally, metabolomics are used to study small molecules and metabolites within cells, biofluids, tissues or organisms [1].

Biological systems are composed of a multitude of molecular entities such as genes, proteins, metabolites and other components and essentially all biological processes are regulated by the interactions among these entities. The analysis of these interactions plays an important part in achieving a mechanistic understanding of physiology and pathology of all forms of life, ranging from single-cell microbes to complex, multicellular organisms. This applies not only at the microscopic scale of the cell interior but also at a macroscopic level; studying the relations between different species occupying an ecosystem can help establish the ecological rules that govern a specific environment. As a result, the study of biological interactions has become a staple of systems biology [2].

As current research involves increasing levels of complexity by combining multiple approaches (e.g., genomics, proteomics, transcriptomics, metabolomics, etc.), particularly in the case of biological interactions [3], the necessity for specialized repositories and advanced integration and visualization techniques emerges. One such technique involves the use of biological interaction networks. In network biology, graphs are often used to represent compartments of whole systems and their biomolecular interactions. A node often represents a biomolecule (e.g., gene, protein, chemical, compound, disease, etc.) whereas an edge the relationship between them (e.g., co-expression, co-occurrence, sequence similarity, coevolution, orthology, homology, fusion, common function, etc.) [4].

Biological interaction networks have been used in a wide range of analyses; some of which have been performed at previously unprecedented scales. The most characteristic example is the Human Interactome Network [5], a proteome-scale analysis of protein–protein interactions for the entire human proteome, which has allowed the detection of previously unknown functional relationships. Starting from an initial analysis for a collection of experimental datasets [6], it is currently a reference map for the human proteome and its interactions, containing more than 50,000 binary interactions featuring almost 90% of the protein-coding genome. Similar genome and proteome-wide interaction networks have also been constructed for a number of other model organisms, such as the mouse (*Mus musculus*) [7,8], yeast (*Saccharomyces cerevisiae*) [9,10] and fruit fly (*Drosophila melanogaster*) [11]. In addition, the combination of gene co-expression and protein–protein interaction evidence with information on metabolic pathways and disease associations has led to the creation and analysis of specialized networks dealing with severe pathological conditions, such as cancer [12], AIDS [13], Alzheimer’s Disease [14,15], and, most recently, infection with SARS-CoV-2, the virus responsible for the COVID-19 pandemic [16].

The successful generation and analysis of interaction networks, such as those referenced above, requires the presentation of information regarding biological interactions in an organized and concise manner. Although this evidence is, to some extent, available in popular biomedical repositories such as PubMed [17], UniProt [18], GenBank [19], or Ensembl [20], their usefulness for the generation of networks is limited, as these databases have not been designed with the analysis of interactions in mind. The aforementioned issue, coupled with the increasing size and complexity of matrices of interactions, as produced by high throughput methods or generated through computational predictions, has led to the emergence of dedicated biological interaction databases. Nowadays, multiple such interaction databases exist [21], acting as specialized repositories of evidence on gene, protein, and small molecule interactions, as well as associations of these interactions with metabolic pathways, host–pathogen relationships, diseases, and even ecological data. However, while the existence of these repositories has provided more immediate access to interaction evidence, their utilization is not always straightforward, as most of these databases are self-contained systems, each containing their own set of interactions and utilizing their unique organization systems and file formats, which are often incompatible with each other [22]. This makes the retrieval, combination, and manipulation of interaction evidence difficult, particularly for new and inexperienced users.

In this review, we outline, organize, and compare biological interaction databases for a number of interaction types, from protein–protein and protein–small molecule complexes to disease-association, host–pathogen and environment-organism interactions. Notably, we do not only focus on the major databases but also on more specialized repositories and we thoroughly present, group, and evaluate most of the publicly available services based on their content, sources of information, data representation methods, and scope. Given the ever-rising wealth of available information on biomolecular interactions and biomedical networks, we aim for this review to reach a broad spectrum of readers varying from experts to beginners, and serve as a reference for the biomedical community.

## 2. File Formats

Before analyzing the various databases, we briefly describe some of the more usual file formats that interaction databases offer. Aiming at a more structured way of storing interaction data, several specific file formats have been introduced. An initial approach was to borrow concepts from graph theory and store a network as an edge list, adjacency matrix, linked-list, or sparse matrix. However, these formats do not allow for metadata storage and, therefore, several other XML-like formats, such as the BioPAX [23], SBML [24], PSI-MI [25], CML [26], and CellML [27] have been introduced. For example, the Systems Biology Markup Language (SBML) is mainly used for biochemical networks and biological processes, the Biological Pathway Exchange format (BioPAX) for biological pathways, the PSI-MI format for data exchange, and the CellML for mathematical models. Notably, both the GraphML [28] format for storing node and edge attributes and the JavaScript Object Notation (JSON) format, which is mainly used for web-based applications, are two of the most widely-used options when building applications. Various databases provide their interaction data in simple text format (tab or comma delimited) or directly in a database schema (e.g., SQLite). To this end, it is worth mentioning that the NDEx [29] is an open-source framework for network sharing. Finally, as each file format comes with certain structural rules, many format-specific parsers (e.g., R/Bioconductor, Biopython) have been implemented and are currently available to facilitate data manipulation.

## 3. Bioentity Interaction Databases

Interaction databases can be categorized based on three main criteria; (i) their type of interactions, (ii) source of information and (iii) data curation [30]. These criteria are also used to organize and describe the bioentity interaction databases presented in the following subsections of this review. The interaction type essentially defines the identity of each database. For example, protein interaction databases describe the physical, and often functional, interactions of proteins with other proteins or small molecules. Similarly, nucleic acid interaction databases contain the interactions of nucleic acids with various other cellular components, while gene co-expression databases describe interactions based on similar gene expression patterns. 

As far as the source of information is concerned, interaction databases can be grouped into three main categories based on their data-acquisition policy. These are (i) primary, (ii) secondary, and (iii) predictive databases [31]. Primary databases directly collect experimental interaction evidence from primary sources, i.e., from scientific publications or from deposited interaction datasets, such as those derived from high-throughput experiments. On the other hand, secondary databases do not collect information from primary sources; instead, they combine and annotate data curated by several primary databases in a single repository. Finally, predictive databases contain not only experimental interaction evidence but also computationally predicted interactions, derived from various methods, such as sequence or structure analysis, or from automatic methods for parsing the literature (e.g., text mining).

The final criterion in classifying interaction databases is their data curation policy; that is, the way the information was collected along with levels of detail and the description, annotation, and classification of this information. Data acquisition can be manual (i.e., handled by curators, or by the scientific community), automated (performed using computational methods), or a combination of the two. As far as the level of curation is concerned, most interaction databases fall between two extremes, lightly or deeply curated. Lightly curated databases aim to publish the maximum amount of interaction information, without necessarily focusing on details. These interactions are often obtained computationally, through automated methods, such as text mining. As a result, lightly curated databases often contain potentially erroneous interactions, as well as redundant or overlapping information. On the other hand, deeply curated databases offer detailed information on biomolecular complexes and the interacting partners involved. This information is periodically manually annotated, validated through multiple sources, and checked for redundancy; the drawback to this manual, detailed approach is that deeply curated databases often contain significantly fewer interactions [30].

Apart from the above, another database aspect that needs to be taken into account is data availability. Two database features pertaining to this aspect are the existence of programmatic access options and the database’s license. Programmatic access refers to the ability to parse a database’s contents programmatically, thus allowing the automated retrieval of multiple entries. Its existence in an interaction database can be very important, especially since the analysis of biomolecular interactions in Systems Biology often involves parsing large amounts of data (hundreds of thousands, or even millions of interactions). Programmatic access can typically be performed through an Application Programming Interface (API), dedicated modules in programming languages such as Python or R, or with extensions/plugins written for external applications (e.g., Cytoscape apps) [32]. As far as the licensing model is concerned, it can depend on various factors, from the data sources of each repository to the policies of the foundations hosting the databases. Generally speaking, most of the databases covered in this review offer free access to their data. In some cases, one of the commonly free access licenses is used (e.g., Creative Commons, GNU/GPL, Apache license etc.), while other databases simply offer their data without adopting a license model at all. However, some databases may impose restrictions, by requiring registration with academic credentials, or by offering only paid access to some of their data. Both the licensing model and the existence of programmatic access are evaluated for the databases presented in this review.

### 3.1. Gene Co-Expression

The key assumption in the construction of co-expression networks is that two genes which are functionally related tend to have similar expression patterns. Hence, poorly characterized genes can be functionally annotated through potentially related genes with similar expression patterns and, as a potential corollary, similar functions [33]. Gene co-expression networks are usually generated by analyzing data from high-throughput gene expression profiling technologies, such as microarrays or RNA–Seq. Normally the co-expression similarity is calculated with the use of metrics such as Pearson or Spearman. In this section, we investigate databases which host such co-expression networks as well as information describing gene-gene relationships across various organisms.

COXPRESdb [34] is a repository that retrieves condition-independent co-expression information from 11 different organisms and focuses on protein-coding RNAs. The major strength of this database is the comparison of multiple co-expression data derived from different transcriptomic technologies (RNA–seq and microarrays) for various species (human, mouse, rat, chicken, zebrafish, fly, nematodes, monkey, dog, budding yeast, and fission yeast). Specifically, the latest update combines gene expression data from 23 different co-expression platforms, of which 123 experiments concern humans, 154 mouse and 154 rat, released by Gene Expression Omnibus (GEO) [35]. In total, COXPRESdb hosts 12 co-expression networks for various species created from ∼157,000 microarray and 10,000 RNA–seq samples. Interspecies comparison reveals the evolutionary relationships, whereas the verification of co-expression interactions from multiple platforms minimizes errors [36]. Additional functionalities include: (i) querying of multiple genes simultaneously, (ii) applying topological network analysis and (iii) module detection.

The Search Tool for the Retrieval of Interacting Genes and proteins (STRING) database [37] (described in more detail in Section 3.3.1) primarily hosts protein–protein interactions for more than 14,000 organisms. However, among the several evidence interaction channels (multi-edged networks), one is dedicated to gene co-expression. The majority of the data for this channel is obtained from transcriptomic technologies as well as proteomic expression data (e.g., ProteomeHD database) [38]. In the co-expression network, every pair of genes with similar expression patterns is scored according to how strong the correlation is. The database offers a number of resources for the analysis of interactions, including a versatile REST API and an interface for Cytoscape [39,40], including a specially designed app (stringApp) [41].

GeneMANIA [42] provides gene co-expression networks and comprises functional gene similarities for nine different organisms (*A. thaliana*, *C. elegans*, *D. rerio*, *D. melanogaster*, *E. coli*, *H. sapiens*, *M. musculus*, *R. norvegicus* and *S. cerevisiae*). It integrates genomics and proteomics data from various sources, such as GEO, the Biological General Repository for Interaction Datasets (BioGRID) [43], IRefIndex [44], and Interologous Interaction Database (I2D) [45]. In GeneMANIA, users can query for closely co-expressed genes among 2300 networks, which consist of approximately 600 million interactions involving ~164,000 genes.

GeneFriends [46], Immuno-Navigator [47] and COEXPEDIA [48] are databases dedicated to gene correlation and transcript expression for *H. sapiens* and *M. musculus*. Specifically, GeneFriends is a tool for inferring gene interactions from co-expression networks, while it provides updated gene and transcript networks based on RNA–seq data from 46,475 human and 34,322 mouse samples. The Immuno-Navigator database gathers cell-type specific gene expression and co-expression data derived from the immune system. Currently, it contains data from 4639 human samples, obtained from 19 cell types from 191 studies, as well as 3434 mouse samples, obtained from 24 cell types from 261 studies. On the other hand, COEXPEDIA focuses on co-expression patterns derived from data from individual studies and which are associated with biomedical information related to anatomy, diseases, and chemicals. At the moment, COEXPEDIA contains 8 million interactions inferred from 384 and 248 GEO studies on humans and mice.

Regarding human tissue-specific co-expression networks, HumanBase [49], HumanNet [50], and Brain gene EXPression (BrainEXP) [51] cover the vast majority of known interactions. HumanBase includes the GIANT web server, which provides human tissue-specific networks via multi-gene queries. The gene associations are obtained from projects such as the Encyclopedia of DNA Elements (ENCODE) [52] and The Cancer Genome Atlas (TCGA) [53]. HumanNet (v2) aims to predict gene co-expression interactions and gene-disease associations through a complex combination of a four-level inclusive hierarchy of the human gene networks. The levels consist of protein–protein interactions, co-functional links from genomics data and two extended functional networks by either co-citations or interologs from other species. Finally, BrainEXP provides data about individual co-expressed genes in normal human brains. It currently stores data from 4567 samples from 2863 healthy individuals.

Various databases focus on plants, especially on *A. Thaliana*; ATTED-II [54], CoP [55], PlaNet [56] and PLANEX [57] cover several plant species, while the Arabidopsis Co-expression Tool (ACT) [58] and AraNet [59] are *A. Thaliana*-specific. The latter two provide co-expression patterns involving 21,273 *A. Thaliana* genes from microarrays and genome-scale functional networks. ATTED-II provides co-regulated gene relationships from microarrays and RNA–seq to estimate gene functions. CoP is focused on biological processes, comprising a microarray-based co-expression network for eight different plant species. PlaNet is a platform which integrates several web-tools dedicated to visualization and analysis of co-expression networks for photosynthetic organisms, while PLANEX is a plant co-expression database, based on publicly available GeneChip [60] data obtained from NCBI GEO. Finally, there are two Algae-dedicated co-expression databases based on Next-Generation Sequencing (NGS) data, ALCOdb [61] and AlgaePath [62], whereas DanioNet [63] is a zebrafish-specific repository. Gene co-expression databases are briefly described in Table 1. Links are summarized in Supplementary Table S1.

### 3.2. RNA and ncRNA Interaction Databases

Non-coding RNAs (ncRNAs) are functionally important due to their interaction with other biomolecules even though they are not translated into proteins. RNA interacting biomolecules may include DNA, other RNAs/ncRNAs, proteins and other chemical compounds, thus influencing various cellular processes. Therefore, in this section, we mainly discuss databases focusing on such RNA interactions.

RNA Bricks2 [66] is a frequently updated database that contains 3D RNA structure motifs and their contact points. It contains more than 4300 RNA structures and RNA–protein complexes originating from the Protein Data Bank (PDB) [67]. RNA network structures are presented as interactive graphs, where nodes depict the basic secondary structure of motifs and edges represent either shared bases or tertiary interactions. RNA Bricks2 contains structure-quality score annotations and offers tools that enable the search of RNA 3D structures and comparisons. It is interconnected with PDB, Rfam [68] and UniProt [18] as the user can browse entries by using identifiers from these databases. Users can query using a FASTA file format, while selected structure data from RNA Bricks2 can be downloaded in PDB format along with a text file that includes a list of interactions. Contact base pairs are annotated through the ClaRNA [69] software and the respective file can be downloaded in CSV format.

As far as ncRNA interaction databases are concerned, NPInter [70] contains functional interactions among various types of ncRNAs (except for tRNAs and rRNAs) and biomolecules such as proteins, RNAs and DNAs. The latest version of NPInter (v4.0) contains a total of 1100,658 interactions, composed by: (i) manually curated literature interactions, (ii) processed high-throughput sequencing data and (iii) interaction data from the RISE [71] database. The interactions concern 35 organisms, while accompanying metadata provide information regarding the interaction class (binding, regulatory, correlation or co-expression) and the tissue/cell line of the experiment, where applicable. NcRNA entries are annotated with identifiers from NONCODE [72], miRBase [73] and circBase [74], while proteins from UniProt [18], Ensembl [20], UniGene (discontinued) and RefSeq [75]. Interactions are downloadable in text format.

Another ncRNA interactions database, snoDB [76], contains manually curated snoRNA interaction data (currently 2089 interactions) from *H. Sapiens*, derived from established databases and literature. SnoDB data are presented in an interactive table view on site and are downloadable in TSV, BED and XLSX file formats. Additional metadata include host genes, species conservation, orthologs and tissue expression where applicable. As snoDB content emanates from various external databases, multiple IDs are used to refer to the same snoRNA with respective links to UCSC [77], RefSeq [75], HUGO Gene Nomenclature Committee (HGNC) [78], Ensembl [20], RNAcentral [79], NCBI, Rfam [68], snoRNAbase (also known as snoRNA–LBME-db) [80], snOPY [81], snoRNA Atlas [82] or RISE [71].

Plant Non-coding RNA Database (PNRD) [83], an updated version of PMRD (plant microRNA database) [84], catalogues plant-related ncRNAs and is currently composed by 25,739 entries, from 11 different ncRNA types across 150 plant species. Nevertheless, its interaction entries regard only miRNAs and their targets, consisting of 178,138 target pairs across 46 plant species. These targets include protein-coding genes, literature ncRNAs and NONCODE lncRNAs and target information has been enriched through psRNATarget [85] and the literature. MiRNA sequence information is mainly derived from miRBase [73] and PMRD [84], while other ncRNAs are mined from NONCODE (v4), Rfam [68], tasiRNAdb [86], GtRNAdb [87], The Arabidopsis Information Resource (TAIR) [88] and the Rice Genome Annotation Project (RGAP) [89]. All database ncRNA sequences are downloadable in text/FASTA format while miRNA–target information and relative literature, in tabular text format. We also note that PNRD hosts a Cytoscape service for constructing miRNA–gene regulatory networks.

Tarbase [90] also focuses on miRNA interactions. It contains manually curated, experimentally supported, miRNA–gene interactions from the literature as well as from raw libraries like GEO and the DNA Data Bank of Japan (DDBJ) [91]. It contains more than 1 million entries that correspond to 670,000 unique, experimentally supported miRNA–target pairs. The interactions within Tarbase are derived from more than 33 high-throughput techniques, applied to 516 cell types and 85 tissues, under 451 experimental conditions, across 18 species. This information is provided as query metadata along with the positive/negative miRNA–target regulation per species and binding locations. Tarbase also incorporates data from miRTarBase [92] and miRecords [93], and supports Ensembl and miRBase identifier queries. The database is interconnected with the Ensembl genome browser and other DIANA-tools, including microT-CDS [94] for in silico identification of miRNA targets, LncBase v2.0 [95] for miRNA–lncRNA interactions identification (see Section 3.2.2) and DIANA-miRPath v3.0 [96] for miRNA functional characterization. Data is available in text format, after filling a request form on the site. The core information of all aforementioned RNA interaction databases is appended on Table 2.

#### 3.2.1. RNA–Protein Interactions

The inherent instability of RNA molecules coupled with the diversity and versatility of their functions are partly responsible for their constant chaperoning by a plethora of different protein complexes. Besides the regulatory binding of proteins to RNA molecules, RNAs also interact with specific proteins to perform specialized functions [97]. Notably, despite the significant contribution of recently developed transcriptome-wide methods and integrative analyses, deciphering the intricate principles of RNA–protein networks is undoubtedly challenging.

In order to facilitate the understanding of these complex, yet vital, interactions, RNA–protein interaction databases integrate experimentally validated and computationally predicted data from published literature and high-throughput technologies, visualizing RNA interactomes [98]. Regarding the contents provided by each resource, RNA–protein interaction databases may be characterized either as comprehensive, incorporating data from multiple sources, specialized, focusing on interactions validated by various experimental methods or predictive, utilizing computational methods, apart from experimental data, to predict possible interactions.

Protein–RNA interaction database (PRD) [99] is a comprehensive database which integrates literature-based physical RNA–protein interactions at the gene level. The current version of PRD contains 10,817 interactions between proteins and protein-coding RNAs, tRNAs, rRNAs, miRNAs, and viral RNAs in 22 organisms, corresponding to 1539 unique gene pairs. Each interaction is enriched with further information curated from multiple other resources, concerning RNA and protein binding sites/motifs, Gene Ontology (GO) [100] terms, detection methods, and biological functions.

The RNA Interactome Database (RNAInter) [101], previously named RAID, is another comprehensive and manually curated database of RNA-associated interactions (RNA–Protein/RNA–RNA), integrating experimentally validated and computationally predicted data from the published literature and 35 other resources. Apart from the fuzzy/batch search, interaction networks, and RNA dynamic expression data that are included in RNAInter, four RNA interactome tools are also embedded, namely, RIscoper [102], IntaRNA [103], PRIdictor [104], and DeepBind [105]. Currently, RNAInter contains 41,322,577 RNA-associated interactions of 22 different RNA types in 154 species, including 34,106,998 RPIs. Identifiers for external databases, such as miRBase, NCBI, HGNC, Ensembl, Online Mendelian Inheritance in Man (OMIM) [106,107], Human Protein Reference Database (HPRD) [108], and UniProt are also provided. Data can be browsed by interaction type, detection method or species and are downloadable in text format, as well as obtainable through an API.

Furthermore, POSTAR3 [109] and doRiNA [110] constitute more specialized repositories, concerning post-translational regulatory RNA–Protein interactions. Both databases provide functional association prediction and contain structural information about binding sites of RNA–binding proteins and RNAs originating from cutting-edge high-throughput sequencing techniques. In particular, POSTAR2 provides the largest collection of RNA–binding protein (RBP) binding sites and functional annotations in 6 species, namely human, mouse, fly, worm, *A. thaliana* and yeast. Three modules (RBP, RNA, and translatome modules) and RBP–RNA interaction network in *H. sapiens* are supported, offering both functional and structural insights into translational and post-translational regulation. On the other hand, doRiNA integrates experimentally validated RBPs and miRNA target site data for *H. sapiens*, *M. musculus*, and *C. elegans*, while computational methods for all species are also used for miRNA target site prediction.

As far as predictive databases are concerned, Protein–RNA Interface Database (PRIDB) [111] contains a total of 30,056 RNA–Protein interactions (5694 unique RNA chains and 1702 unique protein chains) and incorporates structural information facilitating the analysis of RNA–protein complexes and their interface, by providing a user-friendly format. The RNA–Binding Protein DataBase (RBPDB) [112] is a manually curated resource of experimentally observed RNA–binding data for 1171 RBPs in humans, mice, flies, and worms. Finally, RNA binding site DataBase (RsiteDB) [113] is another predictive database aiming to describe, classify, and predict interactions between protein binding sites and single-stranded RNA bases. Table 3 contains information regarding all aforementioned RNA–protein interaction databases.

#### 3.2.2. LncRNA–Target Interactions

Long non-coding RNAs (lncRNAs) are transcripts defined as greater than 200 nucleotides in size, which lack protein coding capacity. LncRNAs play a crucial role in biological processes such as cell cycle regulation, immune responses, and embryonic stem cell pluripotency. Studying lncRNAs is also important in order to understand the underlying mechanisms related to the pathogenesis of various diseases, such as cancer. Here, we evaluate relevant databases that compile and integrate information about lncRNA–target interactions.

LncRNA2Target [114] contains a comprehensive repository of lncRNAs and their target genes regarding *H. Sapiens* and *M. Musculus*, hosting 152,137 associations from 1047 manuscripts (manual literature extraction) and 224 datasets. High-throughput microarray or RNA–seq datasets were used to identify all differentially expressed genes by checking expression before and after knockdown of lncRNAs. All lncRNAs were annotated by NCBI Genbank, Ensembl, GENCODE [115], and Entrez ID/symbols and gene targets by Entrez ID/symbols [116]. Furthermore, each interaction provides a link to the relative publication through a PubMed identifier (PMID). Users can browse and download all lncRNA–target interaction data in text and XLSX format.

EVLncRNAs [117] contains lncRNA interactions validated by low-throughput experiments, such as qRT-PCR, knock-down, western blot, northern blot, and luciferase reporter assays. These interactions are mainly curated from the literature and consist of lncRNA interactions with biomolecules such as DNA, RNA, proteins, and transcription factors (TFs), similar to the RNAInter database, which has already been discussed in the section “*RNA–protein interactions*”. EVLncRNAs also incorporates lncRNA interaction entries from other databases, such as lncRNAdb [118] (discontinued), LncRNADisease [119], and Lnc2Cancer [120] (both discussed below), along with enhanced, manually curated metadata. Its current version (v2.0, July 2020) covers a total of 4010 lncRNAs and 6244 biomolecular interactions across 124 species, and 11,257 lncRNA–disease associations across 1082 diseases. Additional metadata are offered for each entry, such as chromosome position, assembly version, type of interaction (binding, regulation or co-expression), lncRNA class, and validation method. Accession numbers to NCBI and Ensembl, as well as PMID links are provided. EVLncRNAs allows data downloading in XLSX format. In addition, EVLncRNAs provides network visualization of all available interactions on site, as well as links to tools for lncRNA prediction. However, predicted interactions are not included in the database itself.

DIANA-LncBase [95] accommodates experimentally verified tissue and cell type specific miRNA–lncRNA interactions in *H. sapiens* and *M. musculus*. MiRNA–lncRNA interactions are derived from the manual curation of published literature and the analysis of high-throughput datasets. The current version of DIANA-LncBase (v3.0) catalogues ~240,000 interactions regarding ~500,000 entries. Interactions can be retrieved by querying with miRNA or gene names/identifiers (for lncRNAs) from Ensembl, RefSeq, miRBase, and the publication of Cabili et al. [121]. Additional filtering criteria, such as species, cell types/tissues, and methodologies can be applied. A second module in DIANA-LncBase contains information about lncRNAs in different cell types, as well as their subcellular localization, in the nucleus and/or cytoplasm. Queried miRNA–lncRNA interactions and lncRNA expression profiles are downloadable in CSV and JSON format, even though there is no option to download all database interactions.

ChIPBase [122] contains interactions of trans-acting factors, such as TFs, transcription cofactors (TCFs), chromatin-remodeling factors (CRFs), DNA-binding proteins, and histone modifications with various types of RNAs such as miRNA, lncRNAs, and other ncRNAs, from ChIP-seq data across 10 species. ChIP-seq peak datasets of these trans-acting factors are retrieved from GEO, ENCODE, the modENCODE project [123], and the NIH Roadmap Epigenomics Project [124]. All experiments contain metadata regarding cell line/tissue, dataset IDs, and Ensembl IDs for the studied genes. Experiments within ChIPBase can be queried and downloaded in text format (one experiment at a time).

As far as lncRNA–disease association databases are concerned, LncRNADisease [119] is a collection of experimentally and/or computationally validated lncRNA–disease and circular RNA–disease associations. The current version (v2.0) contains more than 200,000 lncRNA–disease and circRNA–disease associations in total, across 4 species. All experimentally supported data are manually retrieved from the literature and the computationally supported data were predicted by four algorithms, LRLSLDA [125], LDAP [126], RWRlncD [127], and LncDisease [128]. Each ncRNA–disease association entry contains detailed information, including gene symbol, gene category, disease information, and regulatory relationship, along with a confidence score. Each disease name is mapped to Disease Ontology (DO) [129] and Medical Subject Headings (MeSH) [130]. All database interactions are downloadable in XLSX format.

Lnc2Cancer [120] is another lncRNA–disease interaction database that focuses on cancer subtypes. The database provides lncRNA–cancer and circRNA–cancer associations, along with their mode of regulation (up or down), supported by experiments. The current version (v3.0) contains 10,303 entries for 2659 human lncRNAs, 743 circRNAs, and 216 cancer subtypes. For every lncRNA or circRNA interaction, flags are provided as additional metadata, relative to their involvement in regulatory mechanisms (miRNA, TF, genetic variant, methylation, and enhancer), biological functions (cell growth, apoptosis, autophagy, EMT, immunity, and coding ability) and clinical applications (metastasis, recurrence, circulation, drug-resistance, and prognosis). lncRNA names are coherent with names from HGNC, Ensembl, GENCODE, Genbank or Refseq, whereas circRNA names are derived from circBase or Circbank [131]. Online data can be browsed by lncRNA/circRNA or cancer names and all interaction data are downloadable in XLSX format.

NONCODE [72] catalogues a variety of ncRNAs, focusing mainly on lncRNAs. NONCODE entries are derived from the literature and the latest versions of several public databases (Ensembl, RefSeq, lncRNAdb and LNCipedia [132]). The bioentity interaction data within NONCODE concern lncRNA–disease associations (32,226) obtained from four lncRNA–disease databases (LncRNADisease, Lnc2Cancer, Mammalian ncRNA–Disease Repository (MNDR—discussed in Section 3.5) [133] and LncRNAWiki [134]) and lncRNA–SNP associations obtained from LincSNP [135] (724,579 total SNPs), which is further discussed below. All entries are accompanied by the respective PMID and each SNP provides a link to dbSNP [136]. NONCODE contains detailed information regarding the sequence, structure, expression, function, conservation, and disease relevance of lncRNAs. All NONCODE sequences are downloadable in FASTA format and all lncRNAs and their respective genes in BED format. However, there is no dedicated download page for the bioentity interaction datasets.

Another lncRNA–disease related database, lncRNASNP2 [137] provides information of SNPs in human and mouse lncRNAs, as well as their impact on lncRNA structure and function. lncRNASNP2 current version (v2) contains 10,205,295 SNPs on 141,353 *H. sapiens* lncRNA transcripts and 5,104,701 SNPs on 117,405 *M. musculus* lncRNA transcripts. lncRNASNP2 transcripts are obtained from 170,002 NONCODE lncRNA genes. lncRNASNP2 also contains predicted lncRNA–miRNA interactions and lncRNA–disease associations. MiRNA sequences were collected from miRBase and disease-associated miRNAs from the Human microRNA Disease Database (HMDD) [138]. Moreover, lncRNASNP2 contains noncoding variants from COSMIC [139,140] cancer data as well as TCGA cancer mutations. All interaction data are downloadable in text format. Online search and prediction tools are also available, enabling the analysis of user-uploaded lncRNAs.

Finally, a similar SNP-centric database, LincSNP [135], stores and annotates disease or phenotype-associated variants, including SNPs, linkage disequilibrium SNPs (LD SNPs), somatic mutations, and RNA editing in human lncRNAs and circRNAs or their regulatory elements. The latter consist of transcription factor binding sites (TFBSs), enhancers, DNase I hypersensitive sites (DHSs), topologically associated domains (TADs), footprints, and open chromatin regions. LincSNP contains entries of experimentally supported variant–lncRNA/circRNA–disease/trait associations retrieved from the literature. LincSNp also incorporates lncRNA information from five databases (Ensembl, LncRBase [135], NONCODE, LNCipedia, and GENCODE). Moreover, disease-associated SNPs were obtained from nine different sources (dbGaP [141], Genetic Association Database (GAD) [142], Gene-wide association study (GWAS) Central [143], Johnson and O’Donnell [144], the National Human Genome Research Institute (NHGRI) GWAS Catalog [145], PharmGKB [146], GWASdb [147], GRASP [148], and LnCeVar [149]). LD-SNPs were collected after analysis by VCFtools [150] and somatic mutations from the COSMIC database [140]. In addition, associations between functional variants, lncRNAs, circRNAs, and their regulatory elements were constructed using BEDTools [151]. Queried interactions are downloadable in spreadsheet, CSV, and PDF formats, while all bioentity site interactions are also downloadable in BED format. All aforementioned lncRNA-related database information is summarized in Table 4.

### 3.3. Protein Interaction Databases

In the following section we discuss databases containing protein interactions. As proteins are responsible for nearly every cell function, the investigation of their interactions is critical to the study of every biological process, as well as the study of diseases and the design of novel pharmaceuticals. As a result, the vast majority of currently available biomolecular interaction databases currently focus on proteins and their interactions, either with other proteins (protein–protein interactions), or with chemical compounds, such as ligands, drugs, and other substances (protein–small molecule interactions).

#### 3.3.1. Protein–Protein Interactions (PPIs)

Proteins rarely act alone inside the cell. Instead, the vast majority of cell functions, from gene expression and metabolic pathways to structural support, cell growth, and cell death, are conducted by multiple proteins, frequently coordinating their action through the formation of protein complexes. Protein–protein interactions are of paramount importance in biological research. Studying the interactions behind a protein–protein complex that conducts a biological process is critical for elucidating the mechanisms that govern that process, as well as for designing better treatments for the diseases that are caused when these interactions are disrupted. For this reason, a significant number of protein–protein interaction databases have emerged in the literature. In this subsection we present a subset of these databases, focusing mainly on repositories that can be of use in Systems Biology, and particularly in the creation and analysis of biological networks.

IntAct [152,153] is a large, open-source, manually curated molecular interaction database hosted by the European Bioinformatics Institute (EBI). All interactions contained in the database are derived from experimental results, obtained from the literature by the database’s curators, or from interaction datasets submitted by the scientific community. IntAct is the largest biomolecular interaction database, as it currently holds more than 11 million binary interactions, the vast majority of which involve protein–protein complexes. In addition to its own data, the database also integrates experimental interaction evidence deposited in MINT [154,155], another major protein–protein interaction database (described in more detail below), as well as interactions derived from UniProtKB/Swiss-Prot and PDB [153]. Each interaction is annotated with details about the experimental procedures followed, as well as accompanied by relevant publications. This annotation evidence is also used to evaluate the confidence of each interaction, by applying a numerical score (Mi-score). Interactions are available for download in the PSI-MI format, both for the entire database and for manually selected datasets, dedicated to specific proteomes or diseases. In addition, database offers a number of resources for the analysis of interactions, including a REST API based on PSICQUIC (Proteomics Standard Initiative Common QUery InterfaCe), an import interface for Cytoscape [39] and a dedicated Cytoscape app (IntAct app) [156] and an embedded network viewer based on Cytoscape-web (a preliminary implementation of Cytoscape.js [157]). IntAct is a major participant in the International Molecular Exchange (IMEx) Consortium, a combined effort to provide an integrative, non-redundant dataset of biomolecular interactions [158].

Similar to IntAct, the MINT (Molecular INTeraction) database [154,155] focuses on experimental evidence derived from peer-reviewed publications. Its data consist of direct (physical) and indirect (functionally inferred) interaction evidence, with each binary interaction entry also containing information on promoter regions, mRNA transcripts, and the functional annotation of its protein partners. Starting from 2014, all interactions deposited in MINT are also integrated into the IntAct database [153]. In addition, MINT has adopted the database organization scheme and infrastructure of IntAct, including the use of the IntAct Mi-score to evaluate data confidence. In contrast to MINT, which exclusively relies on manual curation, the Database of Interacting Proteins (DIP) [159] catalogs experimentally determined interactions that are curated, both manually by expert curators and automatically, using computational approaches. DIP combines information from a variety of sources to create a single, consistent set of protein–protein interactions, each of which is annotated with cross-references to major biological repositories, such as UniProt, RefSeq, and GO. The Integrated Interactions Database (IID) [160] is a database of experimentally detected and predicted protein–protein interactions in 18 species, including human, 5 model organisms, and 12 domesticated species. IID collects experimental evidence from nine PPI databases and combines them with computational predictions using a number of different approaches. Each interaction is annotated with information on the experimental or computational procedure followed, as well as with cell type and tissue expression evidence, where available. In addition, IID offers a number of tools for the creation and topological analysis of PPI networks. MINT, DIP, and IID are all active participants in the IMEx Consortium and utilize the same REST API for programmatic access [158].

BioGRID [43] is a curated biological interaction database, comprising primarily protein–protein interactions, as well as genetic and chemical interactions and post-translational modifications. It strives to provide a comprehensive curated resource for all major model organism species while attempting to remove redundancy to create a single data map. BioGRID is currently one of the largest repositories of biomolecular interactions, containing over 1,740,000 protein–protein interactions curated from both high-throughput datasets and individual focused studies, derived from over 70,000+ publications. Although BioGRID is not an active participant in the IMEx Consortium, it complies with the latter’s guidelines and data format and has been classified as an IMEx Observer. The database provides programmatic access through a REST API, as well as the PSICQUIC API of IMEx members, in addition to integration with the Cytoscape network analysis program.

The STRING database is a large collection of experimentally derived and computationally inferred interactions [37]. STRING is a secondary database (or meta-database), compiling evidence from various sources, including experimental evidence from several primary PPI databases and computationally inferred interactions from literature text mining of scientific texts, de novo prediction of genomic features, and inference based on orthology with model organisms. A major aim of this database is the widest possible coverage of interactions in as many different organisms as possible. As such, STRING currently contains interaction evidence, either experimental or computational, for more than 14,000 species. Each interaction in STRING is annotated as direct/physical or indirect/functional, based on its data sources, and is ranked using a confidence score. STRING provides users with a versatile network visualization platform for the generation and analysis of PPI networks [161], including the analysis of topological features, as well as functional enrichment with terms from GO, KEGG (Kyoto Encyclopedia of Genes and Genomes) [162], Reactome [163], DO, Pfam, InterPro, and the Simple Modular Architecture Research Tool (SMART) [164]. In addition, the database offers programmatic access through a REST API, packages for the R and Python languages, direct integration with Cytoscape and a specially designed Cytoscape (stringApp), capable of building PPI networks with the characteristic STRING visualization style [41].

I2D, formerly known as Online Predicted Human Interaction Database (OPHID), contains protein–protein interactions for a number of mammals and other eukaryotic species [45]. It contains experimental evidence, obtained from high-throughput experiments as well as other databases, such as IntAct or BioGRID, and predicted interactions, inferred by mapping experimental results between different species. In addition, the database implements NAViGaTOR [165], a web-based network analysis platform for the visualization and analysis of PPI networks derived from its data. Although a significant portion of its content has migrated to IID [160], I2D remains one of the most comprehensive sources of known and predicted eukaryotic PPIs for model organisms, such as *S. cerevisiae*, *C. elegans*, *D. melonogaster*, *R. norvegicus*, *M. musculus*, and *H. sapiens.*

The Protein Interactions Network Analysis (PINA) database [166] is an integrated platform for the visualization and analysis of protein–protein interactions through the use of PPI networks. PINA consists of a non-redundant dataset of protein–protein interactions from seven model organisms (*H. sapiens*, *M. musculus*, *R. norvegicus*, *D. melanogaster*, *C. elegans*, *S. cerevisiae*, *and A. thaliana*), obtained from integrating data from five manually curated databases (IntAct, MINT, BioGRID, HPRD, and DIP). The database offers a large number of tools for the construction, visualization, and analysis of PPI networks. In addition, PINA has implemented search and visualization schemes for the analysis of interactions associated with various types of cancer, integrating PPI evidence with RNA–seq transcriptomes and mass spectrometry-based proteomes.

The Compartmentalized Protein–Protein Interactions (ComPPI) database [167] is a large collection of protein–protein interactions from four model organisms (*H. sapiens*, *D. melanogaster*, *S. cerevisiae*, and *C. elegans*). The database currently contains 791,059 interactions, obtained from several other PPI databases and manually curated for redundancy. These interactions are combined with evidence on protein subcellular localization, tissue and cell type expression evidence, and can be utilized to produce tissue-specific, cell-specific, and even subcellular location-specific interaction networks.

CORUM (Comprehensive Resource of Mammalian protein complexes) [168] is a collection of manually annotated protein complexes from mammalian organisms. Its annotation includes protein complex function, localization, subunit composition, literature references, functional enrichment with GO terms, and associations with diseases. All information is obtained from individual experiments published in scientific articles, while data from high-throughput experiments is excluded. For this reason, the total number of interactions in CORUM is relatively small compared to other repositories; however, its data curation for each entry is significantly more detailed. Similar to CORUM, ComplexPortal [169] is a manually annotated and curated resource on macromolecular complexes, with emphasis on protein–protein and, to a lesser extent, protein–nucleic acid, and protein–small molecule complexes. Its interactions are derived from physical molecular interaction evidence extracted and cross-referenced from the literature and deposited in IntAct, by curator inference from information on homologs in closely related species. A key characteristic of ComplexPortal is its strict definition of the term “macromolecular complex” as an assembly of any two or more bioentities that are stable enough in vitro to be reconstituted and have been demonstrated to have a specific molecular function. This means that only constant protein–protein complexes are included in the database, while transient interactions, like those formed in processes such as signal transduction, are discarded. Another key feature of the database is its rich annotation, as each macromolecular complex is accompanied by detailed description of its stoichiometry, function, and relation to biological processes and diseases.

In addition to the major PPI databases described above, a number of web services that focus on specific systems also exist. These include databases which cover the interactions of specialized groups of proteins (or often a single protein class or family) with biomedical or pharmacological interest. Characteristic examples include major drug targets, such as G-protein coupled receptors (GPCRs), Receptor-Tyrosine Kinases (RTKs), and ion channels. A number of databases exist that specialize in describing the features of these proteins, including their interactions. For example, GPCRdb [170] contains both structural and functional evidence on the interactions of GPCRs with ligands and heterotrimeric G-proteins. In the same vein, hGPCRnet (Human GPCR network) provides a network visualization and associated database for PPIs in human GPCR signaling pathways, accompanied with annotation regarding cell and tissue expression [171]. As far as RTKs are concerned, one detailed resource is PrimesDB (Protein interaction machines in oncogenic EGF receptor signalling) [172], which focuses exclusively on PPIs related to the signaling mechanisms of EGFR and ERBB. EGFR and ERBB are two major biomarkers and drug targets in several diseases, including various forms of cancer. PrimesDB also offers tools for the visualization of PPI networks and is a participant in the IMEx Consortium. Finally, Channelpedia [173] is a community-driven database on the features of ion channels, including the interactions between their subunits. All of the aforementioned protein classes and their interactions are also collected and presented in the IUPHAR/BPS Guide to Pharmacology [174], a manually curated dataset of biomolecular interactions implicated in the signaling pathways of human, mouse and rat GPCRs, ion channels, RTKs and other drug targets. Apart from specialization into protein classes/families, databases also exist that provide information on PPIs observed in specific subcellular locations, such as organelles, vesicles, the cell membrane or the extracellular matrix. MitoProteome [175] is a database describing proteins present in mitochondria and their interactions. PerMemDB [176] collects experimental and computationally predicted information on peripheral membrane proteins, including their interactions with transmembrane proteins. Finally, the protein–protein interactions of the extracellular matrix (ECM) are covered by MatrixDB [177], a manually curated database on the PPIs of ECM proteins and proteoglycans. Table 5 presents a collection of available PPI databases.

#### 3.3.2. Protein–Small Molecule Interactions

The interactions of proteins with small molecules are vital for a wide range of biological functions. Inside a cell, small molecules play a twofold role as substrates, cofactors, and products in various biochemical reactions and as ligands or hormones which regulate protein functions [181]. Additionally, bioactive small molecules are often used as probes to identify therapeutic protein targets in drug discovery. Information on the structures, calculated properties, and bioactivities for a large number of chemicals and drug-like compounds is integrated in specialized databases, including PubChem [182], ChEMBL [183], and SIDER [184], with the aim of deciphering their properties and facilitating the drug discovery process. Another essential data resource involves databases focused on protein-chemical interactions, which gather information on the existence, stoichiometry, and biological or biomedical relevance of protein–small molecule complexes [185]. In Table 6, we have collected the relevant information on protein–small molecule interactions databases.

The primary, and most often used source of information in protein-small molecule interactions comes from databases focusing on experimentally studied protein-chemical complexes. DrugBank [186] is currently one of the most popular databases in this category. It is a manually curated and publicly available resource that provides primarily experimental information about small molecules (i.e., chemical, pharmacological, and pharmaceutical) and their protein targets (i.e., sequence, structure, metabolic pathways). In addition to drug-drug interactions, the database incorporates information for physical drug-target interactions and interactions with proteins known to metabolize a compound. Despite its name, however, the database does not focus solely on drugs, but also provides information on other compound types, such as metabolites. DrugBank is a frequently updated resource and its latest release (April 2021) integrates 14,524 drug entries, including 2684 approved small molecule drugs, 1464 approved biologics (proteins, peptides, vaccines, and allergenics), 131 nutraceuticals, and over 6654 experimental (discovery-phase) drugs. Finally, 5249 non-redundant protein (i.e., drug target/enzyme/transporter/carrier) sequences are associated with the aforementioned drug entries.

Another important, experimentally focused protein-small molecule interaction database is BindingDB [187]. BindingDB is a specialized repository of experimentally validated and measured binding affinities between drug-like compounds and therapeutically relevant protein targets. In particular, the latest version of BindingDB incorporates 41,328 Entries, each with a DOI, containing 2,259,122 binding data for 977,487 small molecules, which are mapped to 8516 protein targets. The database is continuously curated, deriving data mainly from scientific articles as well as from US patents. The search interface is well-designed and enables combined query criteria, including target name, sequence, molecular weight, source organism, compound name, SMILES string, binding potency, and article or patent information, while restricted searches by data source (e.g., BindingDB, ChEMBL, PubChem, and patents) is also allowed. 

Apart from the primary databases described above, several secondary repositories also exist, combining information from multiple sources. STITCH (Search Tool for Interactions of Chemicals) [188], the “sister” database of STRING, is a manually curated resource to explore both known and predicted interactions between 9,600,000 proteins from 2031 eukaryotic and prokaryotic genomes and over 430,000 chemicals. Known interaction evidence is mainly derived from experimentally validated data as well as from manually curated datasets, including KEGG and Reactome. Protein–small molecule interactions are also accompanied by protein–protein interaction evidence, derived from STRING, to help illustrate the effect of chemicals on supramolecular assemblies. Text mining-based associations are compiled after parsing articles from PubMed Central (PMC) and PubMed. Like STRING, STITCH offers a REST API for programmatic access, as well as integration with Cytoscape. 

Similar to STITCH, ConsensusPathDB [179] contains human interaction data referring to biochemical reactions and protein, genetic, metabolic, signaling, or drug-target interactions as well as gene regulatory interactions involving different types of physical entities. SuperTarget [189] is another secondary database which hosts information from various databases. It contains 332,828 drug-target interactions along with pathways, protein–protein interactions, and drug-target-related ontologies, based on information retrieved from DrugBank, BindingDB, SuperCYP [190], ConsensusPathDB and CORUM. Metrabase (Metabolism and Transport Database) [191] is another comprehensive cheminformatics and bioinformatics database providing manually curated data extracted from published literature and other resources (TP-Search [192], ChEMBL, Human Protein Atlas [193], DrugBank, and UniProt) related to human metabolism and transport of chemical compounds across biological membranes and their interactions with proteins. Apart from transporter/enzyme-ligand associations, Metrabase incorporates experimentally validated information on non-substrate, non-inhibitor, and non-inducer compounds, aiming to assist the prediction of models based on the characteristics of both the positive and the negative class. Another example is Transformer [194], a database that focuses on the metabolism and transport of chemical compounds in the human body and, more specifically, xenobiotics. It contains integrated data on transformation, transportation, conjugation, and excretion of drugs, prodrugs, alimentary and Traditional Chinese Medicine compounds as well as their effect on enzymes and proteins, also providing the ability of interactive visualization.

A major field of interest in the study of protein–small molecule interactions involves the structural analysis of protein–ligand complexes. A number of specialized databases exist for this purpose. Some of these repositories are, essentially, subsets of PDB, containing analysis on the stoichiometry of protein–heteroatom interactions often found in the PDB entries of experimental 3D structures. PLI (Protein–Ligand Interaction) [195] and PLIC (Protein–Ligand Interaction Clusters) [196] are two such databases, which, as their names indicate, focus on protein-ligand associations. PLI database incorporates all the interactions between proteins and small molecules identified in the PDB with a Het_id code, while PLIC, by analyzing similarities in binding sites and employing computational tools, provides clusters of similar binding sites from PDB. Notably, PLIC, unlike other protein-ligand specific databases, not only reports similarities in interactions but also hosts data on attributes, such as binding site shape, protein–ligand contacts, and energetics among similar protein–ligand interactions. 

In addition to the above, a number of structural databases also exist that complement crystallographic evidence with computational predictions derived from energy calculations, protein-ligand docking predictions or ab initio simulations. NLDB (Natural Ligand Database) [197] is a predictive database focusing on 3D protein-ligand interactions specifically in enzymatic reactions of metabolic pathways registered in KEGG. Based on the latest update, NLDB offers data about known human genome polymorphisms on protein structures, as well as 87,400 experimentally validated protein–ligand complex structures in PDB, defined as natural complexes, while 31,672 analog complexes and 70,570 ab initio complexes were predicted based on known protein structures in a complex with a similar ligand and by docking simulations accordingly. In cases of unknown complex structures, 3D interactions are predicted by implementing state-of-the-art software programs and subsequently generating a database of the 3D protein–ligand interactions in various enzymatic reactions. NLDB also provides an enrichment analysis function based on a set of KEGG compound IDs. PoSSuM (Pocket Similarity Search using Multi-Sketches) [198] is another predictive database that aims to retrieve similar small-molecule binding pockets on proteins with both different and similar global folds, contributing to structure-based drug discovery. It employs the *SketchSort* [199] algorithm for all-pair similarity searches, resulting in more than 163 million similar pairs of binding sites with annotations. Finally, PDID (Protein-Drug Interaction Database) [200] is a database of predicted protein–ligand interactions in the structural human proteome. PDID incorporates 9652 structures from 3746 proteins and provides a comprehensive set of 16,800 putative protein–drug interactions between 51 popular, FDA-approved drugs and over 10,000 protein structures, which were generated from approximately 1.1 million all-atom structure-based predictions.

The databases described above offer generalized information on the existence and properties of protein–small molecule complexes. However, specialized repositories also exist, focusing on the protein–chemical interactions associated with specific systems, phenotypes or diseases. One characteristic example involves cancer-specific databases, such as CancerDR [201], CAncerREsource 2 [202], and canSAR [203]. As their names indicate, these databases focus on protein–drug interactions related particularly to cancer. CancerDR incorporates 148 anticancer drugs which are mapped to 116 drug targets in 1000 cancer cell lines, also offering information about the function, structure, and gene sequences of each of these targets. In addition, CancerREsource 2 contains not only comprehensive data on 90,744 interactions between drugs and cancer-relevant protein targets, but also mRNA expression and non-synonymous mutation data from large-scale cancer genomics experiments. Similarly to the previously mentioned databases, canSAR is a comprehensive database which integrates protein–drug interactions between 564,407 proteins from all species and 3,312,866 compounds with unique chemical structures, as well as genomic and structural data.

Finally, one important category of specialized protein–small molecule databases focuses on the interactions of kinases, a large group of enzymes that participates in a multitude of cell processes and which, as such, has been implicated in a wide range of diseases. Kinase-specific databases include KIDFamMap (Kinase-inhibitor-disease family map) [204] and KLIFS (Kinase-Ligand Interaction Fingerprints and Structures database) [205], which contain protein–chemical information oriented to the kinase superfamily. In particular, KIDFamMap includes 189,987 kinase-inhibitor interactions derived from BindingDB and grouped into 1210 kinase-inhibitor families according to their pharma-interfaces, providing associations between 399 human protein kinases, 35,788 kinase inhibitors, and 339 diseases. KLIFS is another comprehensive kinase database that focuses on the interactions between 3499 kinase inhibitors and 312 kinases, based on the chemical structure of their catalytic domains. Finally, kinase-substrate interactions are also included in the IUPHAR/BPS Guide to Pharmacology [174], a database which, among other drug targets, includes a special section dedicated to the functionality and pharmacology of kinases.

**Table 6:** Protein–small molecule interactions databases.

Database Name	Interaction Type	Data Category	Curation Type	Organisms	Data License ^1^	Programmatic Access
DrugBank [186]	protein–chemical	Primary	Manual	*H. sapiens*	Free for academic users (CC-BY-NC)	REST & SQL API
BindingDB [187]	protein–chemical	Primary	Manual, Automated	*H. sapiens*	Free (CC-BY-SA)	RESTful API
STITCH [188]	protein–chemical	Secondary, Predictive	Automated	2031 eukaryotic and prokaryotic genomes	Free for academic users (EMBL License)	REST API, Cytoscape app [41]
ConsensusPathDB [179]	protein–protein, protein–chemical	Secondary	Manual	*H. sapiens*	Free for academic users	SOAP/WSDL API, Cytoscape app [180]
SuperTarget [189]	protein–protein, protein–chemical	Secondary	Manual	*H. sapiens*	Free (CC-BY-NC-SA)	N/A
Metrabase [191]	protein–chemical	Primary	Manual	*H. sapiens*	Free (CC-BY-SA)	N/A
Transformer [194]	protein–chemical	Primary	Manual	*H. sapiens*	Free	N/A
PLI [195]	protein–chemical	Secondary	Automated	100 species	Free	N/A
PLIC [196]	protein–chemical	Secondary	Automated	All organisms with available protein–ligand structures in PDB	Free	REST API
NLDB [197]	protein–chemical	Secondary & Predictive	Automated	All organisms with available protein–ligand structures in PDB	Free	N/A
PoSSuM [198]	protein–small molecule	Secondary	Manual	*H. sapiens*	Free	N/A
PDID [200]	protein–chemical	Secondary	Manual	*H. sapiens*	Free (Open Source)	N/A
CancerDR [201]	protein–chemical	Primary	Manual	*H. sapiens*	Free	N/A
CAncerREsource [202]	protein–chemical	Primary	Manual	*H. sapiens*	Free	N/A
canSAR [203]	protein–chemical	Primary	Manual	*H. sapiens*	Free (Public Domain)	N/A
KIDFamMap [204]	protein–protein, protein–chemical	Primary	Manual	*H. sapiens*	Free	N/A
KLIFS [205]	protein–chemical	Primary	Manual, Automated	*H. sapiens*, *M. musculus*	Free (Open Source)	REST API
TDR Targets [206]	protein–chemical	Primary	Manual	*11 host organisms*, *35 pathogens*	Free	N/A
T3DB [207]	protein–chemical	Primary	Manual	*H. sapiens*	Free	N/A
BioLiP [208]	protein–chemical	Secondary	Semi-manual	~100 species	Free	N/A
Binding MOAD [209]	protein–chemical	Primary	Manual	>100 species	Free	N/A
ASDCD [210]	protein–chemical	Primary	Manual	*H. sapiens*	Free	N/A
PRRDB 2.0 [211]	protein–small molecule	Primary	Manual	7 species	Free	N/A

^1^ The license type adopted by each database. In cases where a specific license type is used, it is given in parentheses. License abbreviations: CC-BY-SA, Creative Commons–Attribution–Share Alike; CC-BY-NC, Creative Commons–Attribution–Non Commercial; CC-BY-NC-SA, Creative Commons–Attribution–Non-Commercial–Share Alike.

### 3.4. Signaling and Metabolic Pathway Interactions

The interactions between all aforementioned molecules (DNA, RNA, proteins, etc.) cause cascading effects that may consequently affect biological mechanisms and processes through signaling and metabolic pathways. Analysis, processing, and interpretation of the vast and ever-growing amounts of -omics- data has made the implementation of pathway-oriented approaches necessary in most fields in Biology. The complexity of biological processes and their innumerable underlying interactions is most effectively and efficiently conceptualized with the representation and visualization of biological pathways [199]. Herein, we summarize a variety of databases dedicated to signaling and metabolic pathway interactions. Table 7 contains information on the discussed signaling and metabolic pathway interaction databases.

WikiPathways [212] is a manually curated database, launched in 2007 that is continuously updated on an almost daily basis. It is a collaborative platform based on the MediaWiki software, which incorporates customized graphical tools for editing and facilitating the representation of biological pathways and processes. The community has consistently been involved in the construction and revision of the pathway models comprising the database. Wikipathways also incorporates content from a large selection of databases, providing users the ability to query pathways from a variety of fields, such as Renal Genomics, the Reactome database, Diseases, Lipids and Micronutrients, through dedicated thematic sections (portals). The WikiPathways database includes a total of 2958 pathways (April 2021) consisting of proteins, genes, metabolites, and drugs, covering *H. sapiens* along with 29 other species and comprises 46,105 interactions between the represented bioentities. A designated wiki page is ascribed to each pathway, including features such as a pathway diagrams, short analysis, list of references as well as a list of all pathway components. The database content is freely accessible through a browser, an API or a specially designed Cytoscape app [213], and is downloadable in multiple formats, such as: (i) image formats (PNG, SVG, PDF), (ii) gene lists (GMT, Eu.Gene format), and (iii) machine-readable formats (GPML, RDF, BioPAX, XGMML, SBGN, SBML) for further pathway analysis by various tools, such as PathVisio [214] and Cytoscape [39]. Links to other databases are provided for pathway components via the BridgeDb web service [215], such as NCBI, GO, Ensembl, UCSC Genome Browser, UniProt-TrEMBL, WIKIGENES [216], PDB, and IUPHAR/BPS Guide to Pharmacology.

Reactome [163] contains manually curated information derived from 33,453 literature references and in principle constitutes an extended metabolic map of *H. sapiens*. It includes detailed information of cellular processes on a molecular level, visualizing them in coherent data models. Such processes range from transport and DNA replication to signal transduction and intricate metabolic functions. Orthologous molecular reactions are also included for various other species, where applicable. The database (version 76) contains 10,867 human genes, 415 drugs, 1856 small molecules which serve as natural substrates, catalysts or regulators, 11,073 discrete proteins and 13,732 reactions incorporated into 2516 human pathways grouped in 26 superpathways (i.e., immune system, metabolism, diseases). The entities are linked to various databases of the relevant type, such as NCBI, Ensembl, UniProt, KEGG (Gene and Compound), ChEBI [217], PubMed, and GO. Reactome data is downloadable in various formats (DOC, PDF, SBML, SBGN, BioPAX 2, BioPAX 3, OWL, PNG, SVG, JPEG, GIF) and can be queried via an API, as well as through a Cytoscape app (ReactomeFIViz) [218].

KEGG [162], rather than constituting a single database, is an integrated database framework comprising 15 databases which are manually curated and an additional computationally generated one. Among them, KEGG PATHWAY [219] contains biological pathways represented graphically by manually drawn pathway maps, similar to Reactome. Listed entities include molecules, genes, proteins, and pathways, as well as disease genes and drug targets. Within the pathway maps, sequenced genes are linked to higher order functions in the context of individual cells or entire organisms. Such functions are depicted by a web of interactions and chemical reactions, drawn in the format of KEGG pathway maps, BRITE hierarchies, and KEGG modules. KEGG contains 34,042,792 genes, 781,759 pathways and 11,505 reactions pertaining to 545 eukaryotes, 6234 bacteria, and 343 Archaea (April 2021). Links are provided to other databases for bioentities included in the various pathways, such as GO, UniProt, other KEGG Databases, Rhea [220], NCBI, PubChem, CheMBL, KNApSAcK [221], PDB (Chemical Components) while PubMed references are also incorporated. The database provides an API, while the content can be downloaded in multiple formats, such as PNG, RDF and KGML. In addition, multiple Cytoscape apps have been developed, both from the database’s curators and from third-party users, that integrate KEGG data visualization and analysis with Cytoscape [222,223,224].

Similar to the aforementioned databases, CBN (Causal Biological Network) [225] provides over 120 manually curated network models using Biological Expression Language (BEL) [226] integrating over 80,000 literature-based information pieces in order to describe signaling pathways and their biomolecular interactions. More specifically, it showcases the relationships in pathways across a wide spectrum of biological fields in 3 species (*H. sapiens*, *M. musculus*, *R. norvegicus*) using interactive network visualizations. These fields include cell fate, cell stress, cell proliferation, inflammation, tissue repair, and angiogenesis in the framework of the pulmonary and cardiovascular systems. Furthermore, the visualizations incorporate interacting entities, including proteins, DNA variants, coding and non-coding RNAs, chemicals, lipids, and processes (e.g., phosphorylation). Pathway compartments are annotated with metadata regarding species, tissue, and cell type and are also accompanied by their original references in PubMed. The networks can be downloaded in several formats, such as JSON GRAPH, SIF and SVG for further analysis. 

Finally, the INDRA (Integrated Network and Dynamical Reasoning Assembler) database [227] is an automated system for the retrieval of interaction information on bioentities. Based on the INDRA model assembly system, the database aggregates knowledge extracted by multiple machine-reading systems from all available abstracts and open-access full text articles, and combines this with mechanisms from pathway databases. Queries allow searching for genes, chemicals, biological processes and other concepts of interest, and returns a ranked list of relevant interactions and molecular pathways. INDRA sources include the PubMed and PubMed Central literature repositories, as well as a large number of other biological information databases, including DrugBank, BioGRID, CBN and many others. The database can be queried through the IDNRA REST API, as well as through a standalone application implemented in Python.

**Table 7:** Signaling and metabolic pathway interaction databases.

Database Name	Interaction Type	Data Category	Curation Type	Organisms	Data License ^1^	Programmatic Access
WikiPathways [212]	pathway-pathway, intra-pathway biomolecular interactions (proteins, genes, drugs, metabolites)	Primary, Secondary	Manual	30 species	Free (CC0)	REST API, RDF API (SPARQL endpoint), PathwayWidget (embedded iframe), API libraries (R, Java, Perl, PHP, Python), Cytoscape app [213]
Reactome [163]	pathway-pathway, intra-pathway biomolecular interactions (proteins, genes, drugs)	Primary	Manual	16 species	Free (CC0)	REST API, ReactomeFIViz app (Cytoscape app) [218], reactome2py(Python), ReactomePA (R), DiagramJs (JavaScript)
KEGG Pathway [219]	pathway-pathway, intra-pathway biomolecular interactions (proteins, genes, drugs)	Primary	Manual	545 eukaryotes, 6234 bacteria, 343 Archaea	Free for standard & API access, paid license for FTP data access	REST API, KEGGscape (Cytoscape app) [222]
CBN [225]	protein-DNA-variant-RNA–ncRNA–chemical-lipid-process	Primary	Manual	3 species (*H. sapiens*, *M. musculus*, *R. norvegicus*)	Free	N/A
INDRA [227]	pathway-pathway, intra-pathway biomolecular interactions (proteins, genes, drugs)	Predictive	Automated	Any	Free (BSD license)	REST API & Python package

^1^ The license type adopted by each database. In cases where a specific license type is used, it is given in parentheses. License abbreviations: CC0; Creative Commons–Public Domain.

### 3.5. Disease-Related Interactions

Perturbations in signaling and metabolic pathway interactions are often the cause of disease. Various databases contain such biomolecular interactions that are implicated in diseases. In this section, we discuss some of these disease-related databases covering biomolecule-biomolecule, biomolecule-disease and bioentity-disease interactions.

Regarding biomolecule-biomolecule interactions, the CIDeR database [228] contains interactions between disease-related biomolecules (and other bioentities, such as environment and phenotype) mainly for metabolic and neurological disorders. There are currently 109,779 interactions between 12,406 biological entries, derived from 11,341 parsed articles. The information is manually curated and each interaction entry is accompanied by its source PubMed ID and the related disease. CIDeR contains a variety of interaction types, such as expression increase/decrease, co-occurrence, co-localization, processing, phosphorylation, transport, and folding. It also holds information about interacting biomolecules such as genes, proteins, complexes, SNPs, mutations, variants, chemical compounds, ncRNAs, and miRNAs. Finally, CIDeR contains interacting bioentities, such as biological processes, pathways, and phenotypes. Each entry is also accompanied by additional metadata (where applicable) regarding the affected organism, tissue/cell line, and gender. CIDeR provides interconnectivity with the Entrez Gene, KEGG, OMIM, miRBase, GO, CORUM, Mammalian Phenotype Ontology (MPO) [229], and BRENDA Tissue Ontology (BTO) [230]. Interactions can be visualized as an interactive 2D network and downloaded in a CSV or SBML format.

MiRNA SNP Disease Database (MSDD) [231] is another database which comprises human disease-related biomolecular interactions. Similar to CIDeR, its data are derived from the literature and are manually curated. MSDD focuses on disease miRNA–SNP interactions, with accompanying metadata, such as the relevant gene and tissue, SNP position relative to the associated miRNA, its allele, and the dysfunction pattern (increase/decrease). Specifically, MSDD provides 525 associations between 182 human miRNAs and 197 SNPs, regarding 153 genes and 164 human diseases. Information was mined in 2387 articles (last update: June 2017). The site allows the user to download MSDD data in text format, while also offering the choice to limit entries to a selected organ. Annotation information regarding miRNAs is derived from miRBase and SNPs from dbSNP.

Other databases provide direct links of biomolecules to diseases, without specifying direct inter-biomolecular interactions. DisGeNET [232] contains both curated and non-curated automatically mined information regarding disease-gene, disease-variant, and disease-disease associations. DisGeNET receives regular updates and its current version (v7.0) covers 1,134,942 gene-disease and 369,554 variant-disease associations, regarding 30,170 disease entries (UMLS [215]), 21,671 genes (NCBI), and 194,515 variants (dbSNP). Curated gene-disease associations are derived from UniProt, ClinGen [233], Genomics England PanelApp [234], PsyGeNET [235], Orphanet [236], the Human Phenotype Ontology (HPO) [237], and Comparative Toxicogenomics Database (CTD) [238], while curated variant-disease associations from UniProt, ClinVar [239], GWASdb, and the GWAS Catalog. DisGeNET data is downloadable in tab-delimited and SQLite database formats. All interaction data are also accessible programmatically through a REST API, an RDF API, the disgenet2r R package and the Cytoscape application. These programmatic endpoints enable downloading data in JSON, XML and TSV formats, as well as allowing disease ID mapping in UMLS, MeSH, OMIM, HPO, DO, Monarch Disease Ontology (MONDO) [240], NCI [241], and ICD-9 [242] databases. 

EnDisease [243] is a manually curated database of enhancer-disease associations. The EnDisease database contains 535 total associations between 133 diseases and 454 enhancers, extracted from 199 published articles in 11 species. The data are downloadable in text format and represent the chromosomal position of the enhancer, the targeted gene and its UCSC identifier, and the related disease, with a respective entry link to the OMIM database. Additional metadata describe the cell type or mutation (where applicable), as well as the PubMed ID of the extracted association.

MNDR [133] is a frequently updated database that provides curated associations between ncRNAs and diseases along with a confidence score. MNDR data are derived from the literature, established databases as well as from predictive algorithms. Specifically, the current version (v3.1) includes 393,651 miRNA–disease, 295,834 lncRNA–disease, 300,630 circRNA–disease, 13,624 piRNA–disease, and 1573 snoRNA–disease associations, for a total of 1,005,312 associations regarding 1614 disease and 11 mammal species. The database entries are downloadable in text format. MNDR also provides an API to programmatically query associations, searching by ncRNA symbol or ID, disease name or DO/MeSH ID. As far as interconnectivity is concerned, MNDR entries contain an official gene symbol or miRBase ID, as well as DO and MeSH identifiers.

The Nervous System Disease NcRNAome Atlas (NSDNA) [244] is another ncRNA–disease association database that specializes in nervous system diseases. Its current version documents 26,128 associations between 144 nervous system diseases and 8736 ncRNAs, regarding 11 species, where information has been manually curated from 1410 articles. The data can be downloaded in text or spreadsheet format. Accompanying metadata describe the organism, tissue, expression pattern, detection method, target, and potential treatment of the association. Regarding database interoperability, NSDNA miRNA symbols were taken from miRBase, lncRNA from NONCODE and lncRNAdb, siRNA from siRecords [228], snoRNA from snoRNA–LBME-db, and piRNA from piRNABank [229]. The relative PubMed ID is also assigned to each ncRNA–disease association.

Several peptides and proteins have been found to possess an inherent tendency to misfold from their native functional state into intractable aggregates. These aggregates, known as “amyloid fibrils”, have been associated with a diverse group of diseases known as “amyloidoses”; examples include the Alzheimer’s and Parkinson’s diseases, Type 2 diabetes, Creutzfeldt-Jakob Syndrome and many others. AmyCo (the Amyloidoses Collection) [245] is a freely available collection of amyloidoses and other clinical disorders related to amyloid deposition. AmyCo classifies 75 diseases into 2 distinct categories, amyloidoses and other clinical conditions associated with amyloidoses. Each disease is associated with its precursor proteins (causative proteins), co-deposited proteins of amyloid deposits and affected tissues or organs. Database entries are also supplemented with detailed annotation and are linked to MeSH, OMIM, PubMed and UniProt databases.

Finally, there are databases linking bioentities, such as phenotypes, to diseases. The Human Phenotype Ontology (HPO) [237] provides human phenotype-disease associations, along with the implicated genes (where applicable) of each phenotype. HPO data are manually curated entries from the OMIM database. OMIM is a regularly updated, major gene-phenotype association database. The HPO is downloadable in OBO and OWL ontology formats. HPO also allows downloading text files with gene-phenotype and phenotype-gene associations, as found in the OMIM, Orphanet, and DECIPHER [246] databases. Gene entries are accompanied by Entrez Gene IDs. Since 2019, HPO provides a REST API to programmatically query HPO entries based on phenotype terms, diseases or genes.

Lastly, NeuroDNet [247] provides manually curated associations of diseases with genetic risk factors and with network models. These models are graphs containing parsed literature information regarding interactions of genes, proteins, and signaling pathways for a neurodegenerative disease. The database contains genetic risk factors regarding 12 neurodegenerative diseases and 16 total disease models for 8 diseases. Disease model networks are visualized through the Celldesigner [232] software and can be downloaded in SBML format. Disease entries are linked to the OMIM database, genes to NCBI, and proteins to the UniProt database, while association links are provided for each PubMed article reference. Information regarding the discussed disease-related databases is appended in Table 8.

#### Host–Pathogen Interactions

A discrete category of interactions that may lead to a disease concerns host–pathogen interactions. Here, we present bioenity interaction databases focusing on such host–pathogen interactions.

Viruses.STRING [248], an extension of STRING, is a database that contains intra-virus and virus–host PPIs. These annotated PPIs are either physical or functional. Interaction data are derived through text-mining, experimental data from BioGrid, Mint/IntAct [153], DIP, HPIDB [249], and VirusMentha [250], and orthologous relationships from eggNOG 4.5 [251]. As of 2021, Viruses.STRING covers 1,380,838,440 interactions between 2031 organisms and more than 9.5 million viral proteins. The site generates interactive networks of the queried interactions and all node entries are linked to Uniprot. Furthermore, the protein entries are also linked to Ensembl, KEGG, GeneCards [252], and neXtProt [253] databases. The data can be fully accessed and analyzed through a REST API and the Cytoscape STRING app. The generated interaction networks can be downloaded in SVG, TSV, XML, and MFA (multi-fasta) formats. All interaction data files are downloadable in text format and the whole database schema in SQL format.

ViRBase [254] is another viral-host interactions database that, apart from just proteins, mainly focuses on ncRNA interactions. More specifically, it includes manually curated associations between viral ncRNAs (especially lncRNAs and miRNAs) and host ncRNAs or proteins. The database (v2.1) currently consists of 781,476 ncRNA interactions between 93 viruses and 27 hosts, derived from 491 articles. microRNA entries were collected from miRBase, lncRNAs from lncRNAdb and the functional lncRNA database [118], snoRNAs from sno/scaRNAbase [255] and snoRNA–LBME-db [80], whereas ICTVdb (International Committee on Taxonomy of Viruses) [256] records provided virus names and abbreviations. Detailed views of the interaction entries consist of confidence scores, detection methods, tissue/cell line of origin and expression changes, where applicable. Furthermore, data can be queried through an API and are also downloadable in XLSX and text formats.

Another host–pathogen interactions database is TDR Targets [206], a repository on protein–chemical interactions involved in neglected disease pathogens, such as those implicated in tropical diseases like African trypanosomiasis (sleeping sickness) or dengue fever. In its latest version, TDR Targets incorporates experimentally determined and computationally predicted annotations on the chemical compounds and metabolites of pathogens associated with diseases and the drugs utilized in the treatment of these conditions, as well as on the sequence and structure features of the proteins targeted. 

MVP (Microbe Versus Phage) [257] database focuses on interactions between phages and prokaryotes (bacteria/archaea). The database incorporates known viral sequences from NCBI, putative prophage regions in bacterial sequences from NCBI and EMBL, as well as viral and prophage sequences from ICTV published datasets, and metagenomic datasets from EBI. For the detection of putative prophage sequences in bacterial/archaeal genomes, the Phage_Finder tool was used [258]. All the viral sequences (50,782) were clustered based on their sequence similarity, resulting in 33,097 viral groups. Interactions and associations between the prophage sequences and microbes are based on 30,321 published sources, including projects such as Uncovering Earth’s virome [259] and ICTV. All phage clusters and prokaryotes in MVP are provided with NCBI taxonomic IDs and all associations are downloadable in text format, whereas visualized networks can be downloaded in SVG and PNG formats. 

Finally, HoPaCI-DB [260] further zooms in on two bacteria, *P. aeruginosa* and *C. burnetii*, and their host interactions. All listed interacting entries are manually curated and consist of either biomolecules such as proteins, nucleic acids or chemical compounds, or bioentities such as cellular processes, phenotypes or environmental factors. Its current version contains 4443 interactions, regarding 371 entries, mined from 290 articles. Database interactions are presented on site either in tabular format or as graph structures. Entries in HoPaCI-DB are mapped to Entrez Gene, KEGG, OMIM, miRBase, GO or CORUM identifiers, depending on their type, and all interactions are accompanied by a relative PubMed ID. Additional metadata describe the type of interaction (e.g., localization, expression change, phosphorylation, etc.) as well as cell type, cell line, and tissue, where applicable. Database interactions are downloadable in CSV and SBML formats. Table 9 incorporates information regarding the aforementioned host–pathogen interaction databases.

### 3.6. Ecological Interactions

Finally, in a more macroscopic view, interactions can be captured between the different species and their relations (prey, pollinate, parasite, etc.). Data banks that include information about such ecosystem interactions aim to capture biodiversity, as well as key biotic and abiotic factors in environmental processes. The following databases include species interactions and trophic webs.

Global Biotic Interactions (GloBI) [261] is an open source database that contains interactions between living organisms and environmental factors. GloBI interaction data are retrieved both from web resources (data journals and APIs) and from directly contacting authors/data managers and are manually curated. The most recent data (May 2021) include 7,824,407 interaction records between approximately 240,000 species. These interactions comprise species’ relationships, such as predator–prey, pollinator–plant, pathogen–host, parasite–host, and describe 33 different interaction types, such as “*eats*”, “*kills*”, “*interacts with*”, “*parasite of*”. The web interface represents interactions in the form of search widgets, interactive maps, hairballs, and bundle diagrams. The records that contain known taxa are cross-referenced with entries in NCBI, World Register of Marine Species (WoRMS) [262], Integrated Taxonomic Information System (ITIS) and Global Biodiversity Information Facility (GBIF), and the site entries are accompanied by links to Wikidata. Dataset collections with interactions are available in TSV, CSV, RDF formats as well as in sqlite, Darwin Core Archive [263], and Neo4j database formats. Data can also be accessed programmatically through a REST API, as well as through R (rglobi) and JavaScript (eol-globi-data-js) libraries or SPARQL and Cypher queries. GloBI is also integrated in the Encyclopedia of Life (EOL) [264] and Gulf of Mexico Species Interactions (GoMexSI) [265] projects.

The Web of Life [266] is a database similar to GloBI, which contains interactions between animals–plants, plants–plants, and host–plants, and visualizes ecological networks on the web in a coordinate-based system. A key difference with GloBI is that Web of Life only provides an “*interacts with*” type of association. At this moment, Web of Life contains 186 interaction networks, regarding 13,244 animal and plant species, which have been assembled by data from both published and unpublished projects. Other than the name of the species and the respective publications, there are no identifiers linking terms to other databases. All networks are provided as adjacency lists and are downloadable in CSV, XLS, JSON, and Pajek formats.

Another database that contains trophic interactions between ~7000 animals and plants in adjacency matrices, similarly to the Web of Life, is the Food Web (GlobalWeb) [267]. By representing the trophic interactions in a network, it is easier to detect the endangered and invasive species that might result from anthropogenic activities, such as fisheries. Currently, Food Web contains 358 food web graphs (adjacency matrix CSV format) that contain information manually mined from 123 reference papers. Again, no identifiers from other databases are provided.

Finally, a more specialized ecological database, focusing on interactions between bats and plants or other organisms, is Bat Eco-Interactions [268]. It currently (May 2021) contains 13,383 interactions that occur between 479 bat species and 2135 other organisms, mined from 622 peer-reviewed articles. Interaction data are available in CSV format after registration. The database receives regular updates with bat–parasite and ba–mammal interactions, which include taxonomic and location metadata. Table 10 summarizes the interaction data of the four aforementioned databases.

## 4. Data Visualization

Network visualization plays a key role in understanding, communicating, exploring and identifying patterns (e.g., important edges, highly connected nodes or communities) in an interactome. For this purpose, several interactive applications have been implemented and a plethora of review papers have been written [269,270,271,272,273]. Briefly, Cytoscape [39,40], Cytoscape.js [157], Gephi [274], Pajek [275], Ondex [276], Proviz [277], VisANT [278], Osprey [279], Arena3D [280,281], Arena3D^web^ [282], Graphia (Kajeka) [283], NORMA [284], and BioLayout Express 3D [285] are state-of-the-art tools worth mentioning. Similarly, pathway-specific applications include the Pathview [286], BioTapestry [287], PathVisio [214], Interactive Pathways Explorer (iPath) [288], MapMan [289], KEGG [162], Reactome [163], WikiPathways [212] and Pathway Commons [290].

In addition to the aforementioned visualizers, several complementary tools have been implemented for network topological analysis and cluster set comparisons. Typical examples are the Network Analyzer [291], ZoomOut [292], Network Analysis Toolkit (NEAT) [293], NAP [294,295], VICTOR [296] and the Stanford Network Analysis Project (SNAP) [297]. Back-end libraries for network storage and analysis which are worth mentioning are igraph [298], NetworkX [299], GraphViz and Graph-tool [300]. Finally, widely used force-directed layout algorithms [301] for efficient graph drawing include the Fruchterman-Reingold [302], Yifan-Hu [303], Large Graph Layout (LGL) [304] and Kamada-Kawai [305] algorithms.

## 5. Functional Enrichment

In order to annotate clusters within a network and gain insights into the biology of the bioentities which tend to form distinct communities, a functional enrichment analysis is necessary. Briefly, functional enrichment analysis is an approach to identify classes of bioentities (e.g., Gene Ontologies, pathways etc.) in which genes or proteins were found to be over-represented. Among several applications which have been proposed [306,307], tools which are worthy mentioning include: g:Profiler [308], Panther [309], DAVID [310], WebGestalt [311], EnrichR [312], AmiGO [313], GeneSCF [314], AllEnricher [315], aGOtool [316], ClueGo [317], GSEA [318], GOrilla [319], Flame [320], clusterProfiler [321] and NASQAR [322].

## 6. Conclusions

While great efforts have been made in the fields of network biology and biomedical data integration, and despite the numerous databases and repositories for organizing data in a more structured way, a number of challenges remain to be addressed. Scalability is one of the major future challenges. The overgrowth trend to biomedical data has been clear at least since 2015 [323], when it was reported that Twitter was producing 1–17 petabytes of information per year, Astronomy with 1000 petabytes per year, YouTube with 1000–2000 petabytes per year, and Genomics with 2000–40,000 petabytes per year. Due to these orders of magnitude in data accumulation, biomedical repositories need to adjust to the new big-data era and adopt new technologies which can cope with today’s complexity and exponential information growth.

Efficient indexing, compression algorithms for massive volumes of information, and usage of cloud computing and distributed systems would constitute significant enhancements. In addition, despite the plethora of biomedical databases, users (especially less experienced ones) still prefer non-biomedical search engines, such as Google, Bing or Yandex to query biomedical terms. This can mainly be attributed to the poor integration between databases and their inefficient search engines, which often do not allow for any user-friendly flexibility. Some progress has been made in this area with the development of systems that integrate multiple resources in a common framework. Perhaps the most characteristic example is the IMEx Consortium [158], which integrates information from multiple interaction databases (IntAct, MINT, DIP etc.) with additional annotation from other sources (e.g., Mechanobiology [324]), and provides a common API (IMEx PSICQUIC) to retrieve and combine information from all its participants. Another example is the Network Data Exchange (NDEx) [29], which also integrates multiple sources in a unified format and access point. However, these systems support only a limited number of the currently available biomolecule and bioentity interaction databases; instead, the vast majority of the databases presented in this review are isolated systems, with poor APIs, documentation, and data accessibility.

In terms of designs, many of the currently available databases often come with unfriendly or complicated GUIs, thus being unattractive, overwhelming and difficult-to-use. Another important issue is the limited cross-talk between the various repositories (web services) along with the lack of ID conversion tools, which rarely cover a broad enough spectrum of common database identifiers. Among the issues that still remain to be addressed are symbol disambiguation, redundant information across repositories, better literature mining tools (e.g., OnTheFly [325] or INDRA [227]), richer metadata, more accurate name entity recognition techniques to link free text with database records [326,327], utilization of semantics, interoperability, and more frequent/automated updating and maintenance. These tasks will undoubtedly keep bioinformaticians busy in the next few years and their successful tackling promises to offer scientists from all ranks and expertise the necessary tools to successfully navigate the ever-increasing complexity of biological data.

## Figures and Tables

**Table 1:** Gene co-expression databases.

Database Name	Interaction Type	Data Category	Curation Type	Organisms	Data License ^1^	Programmatic Access
COXPRESdb [34]	Gene co-expression	Primary	Manual	11 species	Free (CC-BY)	REST API
STRING [37]	Gene co-expression, Protein–protein interactions	Secondary	Automated	>14,000 species	Free (CC-BY)	REST API, R & Python packages, Cytoscape import & app (stringApp) [41]
GeneMANIA [42]	Gene co-expression	Predictive	Automated	9 species	Free	Command line tool, Cytoscape app [64]
GeneFriends [46]	Gene and transcript co-expression	Primary	Manual	2 (*H. sapiens*, *M. musculus*)	Free	Cytoscape import
Immuno-Navigator [47]	Cell-type specific co-expression, related to immune system	Primary	Manual	2 (*H. sapiens*, *M. musculus*)	Free	N/A
COEXPEDIA [48]	Functional co-expression patterns	Primary	Manual	2 (*H. sapiens*, *M. musculus*)	Free	N/A
HumanBase [49]	Tissue-specific co-expression	Primary	Manual	*H. sapiens*	Free	REST API
HumanNet [50]	Gene co-expression	Predictive	Automated	*H. sapiens*	Free (CC-BY-SA)	N/A
BrainEXP [51]	Brain region co-expression	Primary	Manual	*H. sapiens*	Free	N/A
Human Gene Correlation Analysis (HGCA) [65]	Gene co-expression	Predictive	Automated	*H. sapiens*	Free	N/A
ATTED-II [54]	Co-expressed gene networks	Primary	Manual	9 Plant species	Free (CC-BY)	REST API, RDF API (SPARQL endpoint), Cytoscape import
CoP [55]	Co-expressed gene networks	Primary	Manual	8 Plant species	Free	N/A
PlaNet [56]	Co-expressed gene networks	Primary	Manual	11 Plant species	Free for academic users (Max Planck institution license)	Python standalone, Cytoscape import
PLANEX [57]	Co-expressed gene networks	Primary	Manual	8 Plant species	Free (CC-BY)	N/A
ACT [58]	Co-expressed gene networks	Primary	Manual	*A. thaliana*	Free	N/A
AraNet [59]	Co-expressed gene networks	Primary	Manual	*A. thaliana*	Free	N/A
ALCOdb [61]	Gene Co-expression	Primary	Manual	Microalgae	Free (CC0)	N/A
AlgaePath [62]	Gene co-expression	Primary	Manual	Algae	Free	N/A
DanioNet [63]	Gene associations	Predictive	Automated	*D. rerio*	Free	N/A

^1^ The license type adopted by each database. In cases where a specific license type is used, it is given in parentheses. License abbreviations: CC-BY, Creative Commons–Attribution (BY); CC-BY-SA, Creative Commons–Attribution–ShareAlike; CC0, Creative Commons public domain.

**Table 2:** RNA interaction databases.

Database Name	Interaction Type	Data Category	Curation Type	Organisms	Data License	Programmatic Access
RNA Bricks2 [66]	RNA–(RNA, proteins, metal ions, water molecules, small molecule ligands)	Secondary	Manual	All organisms with available RNA structures in PDB	Free	N/A
NPInter [70]	lncRNA–miRNA–ncRNA–circRNA–vtRNAs-snoRNA–snRNA–sRNA–piRNAs-mRNA–protein-pseudogene-DNA	Primary, Secondary	Manual	35 species	Free	N/A
snoDB [76]	snoRNA–(rRNA, snRNA, non-canonical)	Primary, Secondary	Manual	*H. Sapiens*	Free	N/A
PNRD [83]	miRNA–(protein-coding genes, ncRNAs, lncRNAs)	Primary, Secondary	Automated	46 plant species	Free	Cytoscape service
Tarbase [90]	miRNA–gene	Primary, Secondary	Manual	18 species	Free	N/A

**Table 3:** RNA–Protein interactions databases.

Database Name	Interaction Type	Data Category	Curation Type	Organisms	Data License	Programmatic Access
PRD [99]	RNA–protein	Primary	Manual	22 species	Free	N/A
RNAInter (RAID) [101]	RNA–protein, RNA–RNA, RNA–DNA, RNA–compound, RNA–histone modification	Primary, Secondary, Predictive	Manual	154 species	Free	REST API
POSTAR3 [109]	RNA–protein	Primary	Automated	6 (*H. sapiens*, *M. musculus*, *C. elegans*, *D. melanogaster*, *A. thaliana*, *S. cerevisiae*)	Free	N/A
doRinA [110]	miRNA–protein	Primary	Manual	4 (*H. sapiens*, *M. musculus*, *C. elegans*, *D. melanogaster*)	Free	REST API, Python API
PRIDB [111]	RNA–protein	Secondary, Predictive	Automated	5 (*H. sapiens*, *M. musculus*, *C. elegans*, *D. melanogaster*, *E. coli*)	Free	N/A
RBPDB [112]	RNA–protein	Primary, Predictive	Manual	4 (*H. sapiens*, *M. musculus*, *C. elegans*, *D. melanogaster*)	Free	N/A
RsiteDB [113]	RNA–protein	Secondary, Predictive	Manual	All organisms with available 3D protein-RNA structures in PDB	Free	N/A

**Table 4:** LncRNA–target interaction databases.

Database Name	Interaction Type	Data Category	Curation Type	Organisms	Data License ^1^	Programmatic Access
LncRNA2Target [114]	lncRNA–gene	Primary	Manual	2 (*H. sapiens*, *M. musculus*)	Free	N/A
EVLncRNAs [117]	lncRNA–DNA, lncRNA–RNA, lncRNA–protein, lncRNA–TF, DNA-TF, peptide–protein, lncRNA–disease	Primary, Secondary	Manual	124 species	Free	N/A
RNAInter (RAID) [101]	lncRNA–DNA, lncRNA–RNA, lncRNA–protein, lncRNA–histone modification, lncRNA–compound	Primary, Secondary, Predictive	Manual	15 species	Free	REST API
DIANA-LncBase [95]	lncRNA–miRNA	Primary, Predictive	Manual, Automated	2 (*H. sapiens*, *M. musculus*)	Free	N/A
ChIPBase [122]	(TF, TCF, CRF, DNA-binding protein, histone modification)–(ncRNA, protein, lncRNA, miRNA)	Primary, Predictive	Automated	10 species	Free	N/A
LncRNADisease [119]	lncRNA–disease, circRNA–disease	Primary, Predictive	Manual	4 (*H. sapiens*, *G. gallus*, *R. norvegicus*, *M. musculus*)	Free	N/A
Lnc2Cancer [120]	lncRNA–cancer, circRNA–cancer	Primary	Manual	*H. sapiens*	Free	N/A
NONCODE [72]	lncRNA–disease, lncRNA–SNP-disease	Secondary	Automated	39 (16 animals, 23 plants)	Free (CC-BY-NC)	N/A
lncRNASNP2 [137]	lncRNA–SNP, lncRNA–disease, lncRNA–miRNA	Secondary, Predictive	Automated	2 (*H. sapiens*, *M. musculus*)	Free	N/A
LincSNP [135]	SNP-lncRNA, SNP-circRNA, LD SNP-lncRNA, LD SNP-circRNA, Somatic mutation-lncRNA, Somatic mutation-circRNA, RNA editing-lncRNA, RNA editing-circRNA	Primary, Secondary, Predictive	Manual	1 (*H. sapiens*)	Free (Open Source)	N/A

^1^ The license type adopted by each database. In cases where a specific license type is used, it is given in parentheses. License abbreviations: CC-BY-NC, Creative Commons–Attribution–Non-commercial.

**Table 5:** A collection of protein–protein interaction databases.

Database Name	Interaction Type	Data Category	Curation Type	Organisms	Data License ^1^	Programmatic Access
IntAct [152]	protein–protein, protein–chemical	Primary & Secondary	Manual	1452 species	Free (Apache 2.0)	REST API (PSICQUIC) Cytoscape import & app [156]
MINT [155]	protein–protein	Primary	Manual	669 species	Free	REST API (PSICQUIC) Cytoscape import
DIP [159]	protein–protein	Primary	Manual (primarily) & Automated	834 species	Free (CC-BY-ND)	REST API (PSICQUIC) Cytoscape import
IID [160]	protein–protein	Predictive	Manual	18 (*H. sapiens*, 5 model organisms, 12 domesticated species)	Free	REST API (PSICQUIC) Cytoscape import
BioGRID [43]	protein–protein, protein–chemical, gene co-expression, PTMs	Predictive	Automated (primarily) & Manual	86 species	Free (GNU/GPL)	REST API, Cytoscape import
STRING [37]	protein–protein, gene co-expression	Secondary	Automated	>14,000 taxa	Free (CC-BY)	REST API (dedicated and PSICQUIC), R & Python packages, Cytoscape import & app (stringApp) [41]
I2D [45]	protein–protein	Predictive	Manual	8 species	Free	Cytoscape import
PINA [166]	protein–protein	Secondary	Manual	7 species	FREE (NIH GDS)	RESTful API, Cytoscape app [178]
ComPPI [167]	protein–protein	Secondary	Manual	4 (*H. sapiens*, *D. melanogaster*, *S. cerevisiae*, *and C. elegans*)	Free (CC-BY-SA)	N/A
CORUM [168]	protein–protein	Primary	Manual	3 (*H. sapiens M. musculus*, *R. norvegicus*)	Free (CC-BY-NC)	N/A
ComplexPortal [169]	protein–protein, protein–small molecule, protein–nucleic acid	Secondary	Manual	26 species	Free	N/A
GPCRdb [170]	protein–protein, specializes in GPCR structural complexes	Secondary	Manual	All metazoa with known GPCRs	Free (Apache 2.0)	REST API
hGPCRnet [171]	protein–protein, specializes in GPCR signaling pathways	Secondary	Manual	*H. sapiens*	Free	N/A
PrimesDB [172]	protein–protein, specializes in RTKs	Secondary	Manual	*H. sapiens*	Free (requires registration)	REST API (PSICQUIC)
Channelpedia [173]	protein–protein, specializes in ion channels	Primary	Manual	Mammals	Free (CC-BY-NC)	N/A
IUPHAR/BPS Guide to Pharmacology [174]	protein–protein, protein–chemical	Secondary	Manual	3 (*H. sapiens M. musculus*, *R. norvegicus*)	Free (CC-BY-SA)	REST API
MitoProteome [175]	protein–protein, specializes in mitochondria	Secondary	Manual	*H. sapiens*	Free	N/A
PerMemDB [176]	protein–protein, specializes in peripheral membrane proteins	Predictive	Manual	1009 species	Free	N/A
MatrixDB [177]	protein–protein, specializes in proteins of the extracellular matrix	Primary	Manual	12 model organisms, primary focus on *H. sapiens*	Free (CC-BY)	REST API (PSICQUIC)
ConsensusPath DB [179]	protein–protein, protein–chemical	Secondary	Manual	*H. sapiens*	Free for academic users	SOAP/WSDL API, Cytoscape app [180]

^1^ The license type adopted by each database. In cases where a specific license type is used, it is given in parentheses. License abbreviations: CC-BY, Creative Commons–Attribution; CC-BY-ND, Creative Commons–Attribution–No derivatives; CC-BY-SA, Creative Commons–Attribution–Share Alike; CC-BY-NC, Creative Commons–Attribution–Non Commercial; GNU/GPL, GNU General Public License; NIH GDS, NIH General Data Sharing policy.

**Table 8:** Disease-related databases.

Database Name	Interaction Type	Data Category	Curation Type	Organism	Data License ^1^	Programmatic Access
CIDeR [228]	cellular component–protein complex–disease–drug–environment–gene–protein–mutant–mRNA–variant–ncRNA–miRNA–organism–phenotype-process–protein modification-SNP-tissue-cell line-CpG site	Primary	Manual	22 species	Free	N/A
MSDD [231]	miRNA–SNP	Primary	Manual	1 (*H. sapiens*)	Free	N/A
DisGeNET [232]	disease–gene, disease–variant, disease–disease	Primary	Manual, Automated	1 (*H. sapiens*)	Free (CC-BY-NC-SA)	REST API, RDF API (SPARQL endpoint), R (disgenet2r), Cytoscape
EnDisease [243]	enhancer–disease	Primary	Manual	11 species	Free	N/A
MNDR [133]	ncRNA–disease	Primary, Secondary, Predictive	Manual, Automated	11 species	Free	REST API
NSDNA [244]	ncRNA–disease	Primary	Manual	11 species	Free	N/A
AmyCo [245]	Protein–disease	Secondary	Manual	1 (*H. sapiens*)	Free	N/A
HPO [237]	phenotype–disease	Secondary	Manual	1 (*H. sapiens*)	Free	REST API
NeuroDNet [247]	genetic risk factor–disease, network model–disease	Primary	Manual	1 (*H. sapiens*)	Free (Open Source)	N/A

^1^ The license type adopted by each database. In cases where a specific license type is used, it is given in parentheses. License abbreviations: CC-BY-NC-SA Creative Commons–Attribution–Non-Commercial–Share Alike.

**Table 9:** Host–pathogen interactions databases.

Database Name	Interaction Type	Data Category	Curation Type	Organisms	Data License	Programmatic Access
Viruses.STRING [248]	viral protein–viral protein, viral protein–host protein	Primary, Secondary, Predictive	Automated	2031 (1678 bacteria, 238 eukaryotes, 115 archaea)	Free for academic users (EMBL License)	REST API, Cytoscape app (stringApp) [41]
ViRBase [254]	host/viral ncRNA/protein–host/viral ncRNA/protein (ncRNA = mRNA, miRNA, pseudo-snoRNA, snRNA, lncRNA, rRNA, circRNA, shRNA)	Primary, Predictive	Manual	93 viruses, 27 host organisms	Free	REST API
TDR Targets [206]	protein–chemical	Primary	Manual	11 host organisms, 35 pathogens	Free	N/A
MVP [257]	bacteria–viral clusters, archaea-viral clusters	Secondary, Predictive	Automated	9122 bacteria, 123 archaea, 33,097 viral clusters (phages)	Free	N/A
HoPaCI-DB [260]	gene/protein-process-disease-organism-tissue/cell line-cellular component-phenotype-complex/PPI-drug-ncRNA/miRNA-organism-gene/protein mutant-environment-mRNA/protein variant	Primary	Manual	15 species	Free	N/A

**Table 10:** Ecological interactions databases.

Database Name	Interaction Type	Data Category	Curation Type	Organisms	Data License	Programmatic Access
GloBI [261]	predator–prey, pollinator–plant, pathogen–host, parasite–host	Secondary	Manual, Automated	644,512 known taxa, 77,245 unknown taxa	Free (Open Source)	REST API, R (rglobi), JavaScript (eol-globi-data-js), SPARQL and Cypher endpoints
The Web of Life [266]	host–parasite, plant–herbivore, food webs, anemone–fish, plant–ant, pollination, seed dispersal	Primary	Manual	13,244 animals/plants	Free	N/A
Food web [267]	fish–animal type–detritus–plankton	Primary	Manual	~7000 animals/plants	Free	N/A
Bat Eco-Interactions [268]	cohabitation–consumption–host–pollination–prey–predation–roost–seed dispersal–transport–visitation bat/living organisms	Primary	Manual	484 bat species, 2146 other species	Free	N/A

## Data Availability

Not applicable.

## Supplementary Materials

The following are available online at https://www.mdpi.com/article/10.3390/biom11081245/s1, Table S1: List of web addresses for the databases presented in this review.
